# Transdisciplinary perspectives for health systems science

**DOI:** 10.1038/s44401-024-00002-3

**Published:** 2024-12-05

**Authors:** Jiang Bian, Nan Liu, Shauna M. Overgaard, Rema Padman, Peter A. D. Steel, Victoria Tiase, Clifford Alan Whitcomb, Jiajie Zhang, Yiye Zhang

**Affiliations:** 1 College of Medicine, University of Florida, University of Florida Health, Gainesville, FL USA; 2 Duke-NUS Medical School, National University of Singapore, Singapore, Singapore; 3 Mayo Clinic, Rochester, MN USA; 4 Heinz College of Information Systems & Public Policy, Carnegie Mellon University, Pittsburgh, PA USA; 5 Weill Cornell Medicine, New York-Presbyterian Hospital, New York, NY USA; 6 School of Medicine, University of Utah, Salt Lake City, UT USA; 7 College of Engineering, Cornell University, Ithaca, NY USA; 8 McWilliams School of Biomedical Informatics, University of Texas Health Science Center at Houston, Houston, TX USA

## Abstract

Health systems science uses systems thinking as part of a transdisciplinary approach that transcends traditional disciplinary boundaries. It integrates and synthesizes knowledge from multiple disciplines to address real-world problems in healthcare with pragmatic solutions. This editorial defines health systems from the perspectives of systems thinking, science, and engineering, discusses their current challenges and opportunities, and envisions how health systems science as a field can advance the continuous learning and improvement of health systems.

Health systems vary in structure and scope across different regions and countries, shaped by legal frameworks and specific institutional arrangements such as regulatory agencies, healthcare governance bodies, and funding mechanisms^1^. The World Health Organization defines health systems as “all the activities whose primary purpose is to promote, restore or maintain health.”^2^ There are regional variations, such as those by the European Observatory on Health Systems and Policies^3^, Asia Pacific Observatory on Health Systems and Policies^4^, and the Agency for Healthcare Research and Quality in the United States^5^. Noting the variations in global health systems definitions, this editorial examines health systems through systems thinking, science, and engineering perspectives^6^.

Unlike software or hardware systems, humans - healthcare service providers, patients, and community members - comprise the health system, thus making it a socio-technical system^7^. While engineers can optimize a purely technical system by applying advanced algorithms to configure their parts, the objective functions for a socio-technical system – especially the human component and particularly those suffering from physical and mental illness – are multifaceted. The fragility and emotions of humans make health systems complex and distinguish them from other systems. Studying health systems involves understanding human behaviors and interactions – from clinicians to patients, patients to caregivers, among clinicians themselves, and hospitals to community members - and examining how societal and environmental changes influence these dynamics.

As key societal structures responsible for population health, health systems inherit complex challenges like aging populations, disparities and inequity, volatile world events, and climate change. These challenges necessitate health systems to look beyond biological mechanisms to consider human behaviors and social determinants of health, and beyond the hospital walls to engage with the communities they serve. Addressing these needs mandates health systems to work with public health agencies and payors to make transformative changes in healthcare delivery, resource allocation, and financing models, all while maintaining standards of care and patient safety. Many health systems have also joined forces to form networks of diverse healthcare institutions, accelerating research^8^.

Attempting to meet these responsibilities has earned health systems the title of heroes, but increasing workforce burnout has placed a greater focus on sustainable solutions. To address this, health systems have begun embracing technology as a potential solution. For example, some have implemented telehealth and deployed predictive models to enhance clinical decision-making, AI scribes to reduce the documentation burden, and chatbots to assist with patient scheduling and messaging. However, implementing new solutions, especially in healthcare, is as much a human challenge as a technical problem. The latest technology, generative AI, also poses ethical and equity questions, as many health systems scramble to understand how to integrate it effectively as a part of healthcare workflows, balancing opportunities with unintended consequences. Thus, health systems must navigate the regulatory and legal implications of the fast-changing technology landscape, while making business decisions in the face of uncertainty about long-term returns on their technology investments. Fortunately, there is a wealth of experience in adopting and influencing new policies and programs from which health systems can draw, such as the Hospital Readmissions Reduction Program^9^ and the Meaningful Use Program^10^. As the evolution of AI sweeps through society, leading health systems and coalitions^11,12^ can serve as testing beds and examples for innovative and responsible co-development and use of AI, while closely examining the changes in human behavior and interactions, care and payment models, and workforce needs around this rapidly changing technology.

The evolution of a health system’s operations and management has significant and far-reaching implications for healthcare ecosystems, diverse patient populations, and society. In acknowledgment that no single discipline alone can sustain and facilitate the continuous learning and improvement of health systems, organizations such as the American Medical Association^13^, the US National Academies of Sciences, Engineering, and Medicine^14^, and the WHO^2^ promote *systems thinking principles* in managing, studying, and teaching health systems. Under systems thinking, knowledge from medicine, health policy and economics, behavioral science, translational and clinical research, informatics and data science, operations research, education, and ethics converge and synthesize practical solutions to address complex, real-world problems for health systems. Such transdisciplinary perspectives among researchers, health system stakeholders, policymakers, and community members will serve as a comprehensive framework for studying health systems science. Additionally, these insights and partnerships will assist health system leaders as they anticipate and navigate the evolution of health systems.
